# The effect of selective ultrasound screening on the incidence of late presentation of developmental hip dysplasia—a meta-analysis

**DOI:** 10.1007/s00247-023-05666-x

**Published:** 2023-04-26

**Authors:** Lene B Laborie, Karen Rosendahl, Amira Dhouib, Paolo Simoni, Paolo Tomà, Amaka C Offiah

**Affiliations:** 1 Section for Pediatric Radiology, Department of Radiology, Haukeland University Hospital, Jonas Lies vei 65, 5021 Bergen, Norway; 2 Department of Clinical Medicine, University of Bergen, Bergen, Norway; 3 Department of Clinical Medicine, Faculty of Health Sciences, UiT the Arctic University of Norway, Tromso, Norway; 4 Section of Paediatric Radiology, University Hospital of North Norway, Tromso, Norway; 5 Department of Radiology, Reseau hospitalier Neuchatelois, Neuchatel, Switzerland; 6 Paediatric Imaging Department, ‘Reine Fabiola’ University Children’s Hospital, Université libre de Bruxelles, Brussels, Belgium; 7 Department of Imaging, Ospedale Pediatrico Bambino Gesù IRCCS, Rome, Italy; 8 Department of Oncology and Metabolism, University of Sheffield, Sheffield, UK; 9 Department of Radiology, Sheffield Children’s NHS Foundation Trust, Sheffield, UK

**Keywords:** DDH, Developmental hip dysplasia, Infant, Late presentation, Meta-analysis, Neonate, Newborn, Screening, Ultrasound

## Abstract

Different screening strategies for developmental dysplasia of the hip (DDH) exist. Despite screening efforts, cases of late presentation continue to occur, often necessitating surgery. This systematic review and meta-analysis assess the effect of newborn selective ultrasound screening for DDH on the incidence of late presentation in infants and children, compared to a universal ultrasound strategy. A systematic search across Medline and EMBASE databases was performed between January 1950 and February 2021. A consensus-based evaluation of abstracts led to retrieval of relevant full text, original articles or systematic reviews in English only. These were assessed according to agreed eligibility criteria, and their reference lists were reviewed to identify additional eligible publications. Following final consensus on included publications, data was extracted, analysed and reported as per PRISMA and Prospero (CRD42021241957) guidelines. The 16 eligible studies consisted of 2 randomised controlled trials and 14 cohort studies, published between 1989 and 2014, with a total of 511,403 participants. In total, 121,470 (23.8%) received a neonatal hip ultrasound, of whom 58,086 and 63,384 were part of a selective or a universal ultrasound screening strategy, respectively. The difference in the proportion of late presentation between the universal and selective strategies was 0.0904 per 1,000 (*P* = 0.047). The time effect, i.e. the difference between early and late presentation defined respectively, as less than and more than 3 months of age, regardless of screening strategy, was not significant (*P* = 0.272). Although there was variability in study design and reporting, the quality of the evidence, based on the critical appraisal skills programme appraisal tools, was generally good. Compared to universal ultrasound screening for DDH, selective screening resulted in a slightly higher rate of late presentation. Uniformity in design and reporting of DDH studies and a cost-effectiveness analysis are needed.

**Supplementary Information:**

The online version contains supplementary material available at 10.1007/s00247-023-05666-x.

## Introduction

Developmental dysplasia of the hip (DDH) in newborns consists of a poorly developed acetabulum and/or an unstable femoral head moving outside of its confined position within the acetabular cup. The diagnosis of DDH can be challenging. The pathomechanical definition of DDH varies, and the choice of method used to confirm DDH varies. A clinical examination of the hip joints is usually performed in all newborns, in many institutions also accompanied by a hip ultrasound (US). Early detected, uncomplicated cases of DDH are successfully treated with an abduction splint in the first months of life. More complicated cases and late detected cases of DDH necessitate longer periods of treatment and often surgical interventions [1]. Complications following untreated DDH include pain, decreased function and development of osteoarthritis in early adulthood [2]. Screening for DDH in the neonatal period is common, and different screening strategies for DDH exist. In addition to clinical hip screening, US can be offered to the approximately 15% of newborns with risk factors (i.e. selective US strategy), or to all newborns (universal US strategy). Despite screening efforts, cases of late presentation continue to occur. An extensive literature related to the most appropriate DDH screening protocol in newborns exists, and the topic continues to be debated. Several systematic reviews performed over the past two decades have reported lack of clear evidence regarding a recommended screening strategy for DDH in the neonatal period [3–5]. The lack of consensus on the most effective screening strategy for DDH is unfortunate. This systematic review and meta-analysis assessed the effect of newborn selective US screening for DDH on the incidence of late presentation in infants and children, compared to a universal US strategy.

## Materials/methods

### Research question and research protocol

We formulated the following research question: What is the effect of selective US screening for DDH on the incidence of late presentation?

The population included newborns from the day of birth and up to 6 weeks of age. The intervention was defined as selective US screening in addition to clinical hip screening, meaning that only those with risk factors (breech presentation at birth, a positive family history of DDH, foot deformities, clinical hip instability) received a hip US in the neonatal period. The comparator was defined as universal US screening in addition to clinical hip screening, meaning that all newborns and babies up to 6 weeks of age received a hip US, regardless of risk factors and clinical findings. The outcome of the study was defined as the incidence of late presentation of DDH in both groups. The definition of ‘late presentation’, as defined by the authors of individual studies, differed. For each study, the age at which ‘late presentation’ was defined, from 4 weeks of age and beyond, was always after the initial newborn screening period.

The research protocol for this systematic review and meta-analysis was registered in the PROSPERO database (CRD42021241957), on 10/03/2021. The study started on 02/02/2021 and was conducted by six paediatric radiologists based in five European countries, on behalf of the musculoskeletal task force of the European Society of Paediatric Radiology (ESPR).

### Inclusion and exclusion criteria

Inclusion and exclusion criteria for the studies were defined prior to the execution of the literature search. Original retrospective and prospective diagnostic accuracy studies were included. Inclusion criteria for study populations comprised newborns, neonates and infants from the day of birth up to 6 weeks of age. Studies with neonates/infants with underlying congenital disorders, including cerebral palsy and other neurological conditions, were excluded. Studies reporting on outcomes from selective and/or universal US screening for DDH were included. Method of screening had to be clearly stated and studies where results for universal and selective screening were not presented separately were excluded.

Original, peer-reviewed retrospective or prospective diagnostic accuracy studies and randomised controlled trials (RCTs) with full text were included in the final study selection. Studies were excluded if they were systematic literature reviews, interventional studies, single case reports, editorials/commentaries or clinical guidelines; if they lacked full text; if they lacked relevant data on screening method and/or outcomes; or if they were not in English. When multiple studies reported findings from the same population, we selected only the most relevant study based on date, sample size and reported analysis of data.

### Literature search and study selection

A systematic search of the literature was performed using PubMed, across the databases EMBASE and MEDLINE, from January 1950 through February 2021 (Supplementary Material 1). The search was performed on March 9, 2021, by librarians at the University of Sheffield/Sheffield Children’s NHS Foundation Trust. No restrictions were applied in the initial search. This review was carried out following the ‘Preferred Reporting Items for Systematic reviews and Meta-Analyses, PRISMA’ [6]. The retrieval was a combination of subject words and free words and the search terms included hip dislocation, congenital; developmental dysplasia of the hip; developmental hip dysplasia; neonatal screening; newborn screening; mass screening; late presentation; early diagnosis; late sequelae; delayed diagnosis; and undiagnosed diseases. One author (A.C.O. with19 years of experience in paediatric radiology) screened all titles and removed those not in English or clearly ineligible. Thereafter, A.C.O. and the other five authors (L.B.L., K.R., A.D., S.P., T.P. with 5, 35, 14, 14, 46 years of experience in paediatric radiology respectively) independently screened titles and abstracts and scored for full text retrieval as eligible or ineligible. A consensus-based evaluation of these abstract scores led to retrieval of relevant full text, original articles or systematic reviews. These were assessed according to agreed eligibility criteria and their reference lists were reviewed to identify additional eligible publications. A consensus-based evaluation of all the full texts proposed for inclusion led to the final study selection.

### Quality assessment of evidence

The quality of each selected study was evaluated using the critical appraisal skills programme (CASP) tools for RCTs and cohort studies [7]. The CASP tools assess the validity of study design and whether studies are methodologically sound. There are 12 questions for cohort studies (Supplementary Material 2) and 11 questions for RCTs (Supplementary Material 3. Each included study was independently evaluated by three authors (each author reviewed 8 papers, and none had performed the data extraction for the studies assigned to them). All the CASP reports were collected by an individual, not otherwise participating in the study, and only shared with all six authors (at the same time) once all assessments had been received.

### Data extraction

From the included studies, the six authors independently extracted relevant data from each study and populated a project-specific spreadsheet (Supplementary Material 4). Discrepancies between classifications or values were discussed and resolved between the extractors. Extracted data included title; author; year; country performing the study; type of study; subject characteristics (number, sex); age of child at time of screening (in days, weeks); ultrasound method employed; risk criteria used to assess eligibility to the selective ultrasound strategy (family history, breech, foot deformities, clinical findings, other); definition of late-diagnosed DDH (when (weeks), how (US, radiography [type of measurement], clinical)); results: number/rate of late-diagnosed cases (including age), of treated cases, of surgery cases and of avascular necrosis cases; number of late detected cases in which initial selective ultrasound was performed; missing data; and total years of follow-up. For publications comparing different screening strategies, data were extracted separately for each strategy.

### Statistical analysis

Descriptive statistics was used to summarise the qualitative data from the 19 studies in the 16 papers. A meta-analysis was used to compare the effects of newborn selective and universal US screening for DDH on the incidence of late presentation in infants and children. First, the meta-analysis tested whether the proportion of late presentation was significantly different from 0.0 (zero) for each of the strategies. Thereafter, it tested the difference between the two strategies. Second, the meta-analysis tested the time effect, i.e. the difference between late presentation defined as less than and defined as more than 3 months of age, regardless of screening strategy. The model heterogeneity (*I*^2^) statistic was applied to determine the percentage of total variation across studies due to heterogeneity [8]. The value of *I*^2^ ranges from 0 (no observed heterogeneity) to 100% (maximal heterogeneity), where *I*^2^ values around 25, 50 and 75% represent low, moderate and high heterogeneity, respectively [9]. The *Q* test is commonly employed to assess the homogeneity of effect sizes, with corresponding degrees of freedom (df). If the *P*-value of the *Q* test is less than 0.05, then the heterogeneity is considered statistically significant. Proportional random-effects (RE) meta-analysis was used to estimate effect size (overall proportion) using the transformed scale using Arcsine-based transformations. This is common procedure for stabilising the variance of proportion in meta-analysis methods, a statistical technique used in meta-analysis to adjust for the variability of proportions (or rates) across different studies. The back transformation was then applied to the transformed effect size to retrieve the original effect size (overall proportion), which can be converted to an incidence rate per 1,000 children. Forest plots were applied to visualise the pooled estimates from the meta-analysis. The point estimate for risk ratio is represented by a black square. The confidence interval (CI) for each study respectively is represented by a thin horizontal line. The publication bias was tested using Rosenthal’s method, also known as a ‘file drawer analysis’. The Statistical Software R version 4.2.2 (R Core Team 2022, Vienna, Austria) was used to perform the random effect model meta-analysis. A *P*-value of <0.05 was considered statistically significant.

## Results

### Qualitative synthesis: characteristics of studies included

**Fig. 1:**
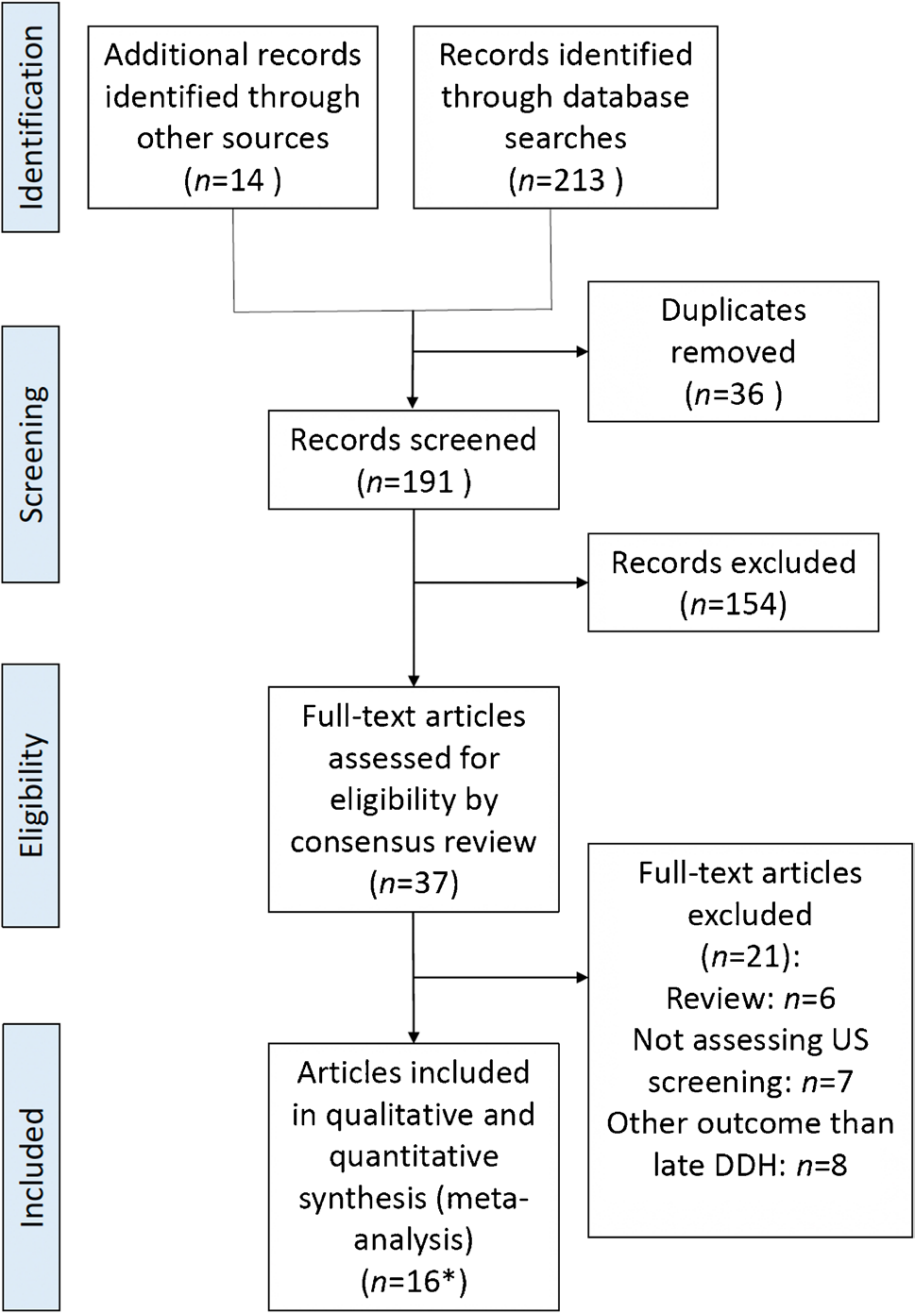
PRISMA flow chart for the article selection and inclusion process. *16 articles were included, representing 19 study periods

The initial search yielded 213 abstracts (Medline = 85, EMBASE = 128). We performed a consensus review of the 23 full text articles retrieved and identified 14 additional full text articles. Data from the 16 eligible publications was extracted (Fig. 1).

The 16 eligible full texts consisted of two RCTs and 14 cohort studies (Table 1) [10–25]. Of the 14 cohort studies, 10 reported on a selective US strategy, 3 on a universal US strategy and 1 on both strategies (performed over two periods of time, thus listed twice [22]). The 2 RCTs were reported on both selective and universal US strategies (both also listed twice) [20, 21]. A total of 19 study periods are therefore examined. Nine of the studies were performed in the UK [10–15, 19, 22, 23]; 3 in Norway (including both RCTs) [16, 20, 21]; and 1 study each in Northern Ireland [17], Scotland [18], Germany [24] and Austria [25]. The studies were published between 1989 and 2018, reporting on data collected during 19 overlapping periods between 1986 and 2014.

**Table 1:** Characteristics of the included studies

First author, year, type of study	Study country and period	Screening	Total population in study period*	Total number screened with ultrasound	Selective screening rate (%)	Rate of abduction treatment (per 1,000)	Rate of surgery (per 1,000)	Author definition of late presentation	Rate of late presentation (per 1,000)
Clarke et al. (1989), [10]. Observational cohort	UK, 1986	S	4,617	448	9.7	3.7	0.68	>2 months	0.68
Boeree et al. (1994), [11]. Prospective cohort	UK, 1988–1992	S	26,952	1,894	7.0	4.4	0.37	>12 weeks	0.22
Lewis et al. (1999), [12]. Prospective cohort	UK, 1988–1992	S	17,792	2,683	15.1	8.1	0.17	NS	0.45
Paton et al. (1999), [13]. Prospective cohort	UK, 1992–1997	S	20,452	1,107	5.4	NS	0.58	>6 months	0.4
Afaq et al. (2011), [14]. Prospective cohort	UK, 2005–2006	S	5,772	200	3.5	1.0	NS	NS	NS
Clarke et al. (2012), [15]. Prospective cohort	UK, 1988–2008	S	107,440	20,344	18.9	7.2	0.74	>12 weeks	0.34
Laborie et al. (2013), [16]. Prospective cohort	Norway, 1991–2006	S	81,564	11,539	13.7	30	0.38	4 weeks	0.32
Donnelly et al. (2015), [17]. Retrospective cohort	Northern Ireland, 2008–2010	S	75,856	NS	NA	8.5	0.54	>1 year	0.42
Tyagi et al. (2016), [18]. Retrospective cohort	Scotland, UK, 2014	S	3,618	428	11.8	3.3	0	>3 months	0.27
Talbot et al. (2017), [19]. Prospective cohort	UK, 1997–2011	S	64,670	NS	NA	NS	0.25	>3 months	0.28
Rosendahl et al. (1994), [20]. RCT	Norway, 1988–1990	U and S	11,925	3,616 (U) 518 (S)	11.8 (S)	34 (U) 20.2 (S)	0 (U) 0.23 (S)	>1 month	0.3 (U) 0.7 (S)
Holen et al. (2002), [21]. RCT	Norway, 1988–1992	U and S	15,529	7,489 (U) 872 (S)	11.3 (S)	9.6 (U) 8.6 (S)	0 (U) 0.01 (S)	>1 month	0.13 (U) 0.65 (S)
Westacott et al. (2018), [22]. Retrospective cohort	UK, 2005–2012	U and S	28,068	10,015 (U) 18,053 (S)	NA	7.9 (U) 2.3 (S)	0.9 (U) 0.6 (S)	>3 months	0.5 (U) 0.28 (S)
Marks et al. (1994), [23]. Retrospective cohort	UK, 1989–1992	U	14,050	14,050	NA	2.4	NS	NS	0
von Kries et al. (2003), [24]. Retrospective cohort	Germany, 1997–2002	U	495	406*	NA	NS	0.26	NS	NS
Biedermann et al. (2018), [25]. Prospective cohort	Austria, 1998-2014	U	28,092	27,808**	NA	10	0.86	NS	0

NA, not applicable; NS, not stated; RCT, randomised controlled trial; S, selective; U, universal; UK, United Kingdom

*89 newborns excluded due to identification outside the routine screening programme

**284 newborns excluded due to various reasons (first hip ultrasound performed after 6 weeks; referral from other hospital; hip-related syndromes; missing baseline data)

The sixteen studies included in the qualitative synthesis and in the quantitative synthesis/meta-analysis had a total of 511,403 participants. Of these, 121,470 (23.8%) received a hip US in the neonatal period, of whom 58,086 (47.8%) were part of a selective and 63,384 (52.2%) were part of a universal US screening strategy, respectively (Table 1).

At clinical hip screening, the age ranged from 0 to 7 days. In 11 of the 16 papers, the age at initial clinical screening was not stated. Nine of the papers stated the clinical exam as Barlow/Ortolani method [11, 13–16, 19–21, 24], whereas 7 papers did not specify the type of clinical test performed [10, 12, 17, 18, 22, 23, 25].

Age at US hip screening ranged from 0 to 6 weeks. Age range was not stated in 10 out of 16 papers. The US method was stated as Graf/modified Graf (Rosendahl) [26, 27] in 8 papers [12, 13, 16, 19, 20, 22, 24, 25], as Clarke’s method [28] in three papers [10, 11, 15], as Harcke’s method [29] in 2 papers [14, 23] and as Terjesen’s method [30] in 1 paper [21]. The US method was not stated in 2 of the papers [17, 18].

Risk factors warranting selective US included abnormal/equivocal clinical findings, positive family history, breech, foot deformities, oligohydramnios, clicking hip, sacral dimple, multiple pregnancy, decreased abduction, ‘various’ and ‘others’. In 6 of the 12 studies using a selective screening strategy, the number of patients within each category was not documented [14, 15, 17–19, 22].

The duration of the study follow-up after neonatal US screening varied from 22 to 5.5 years. Durations of 22 weeks [10]; >6 months [18]; a minimum of 2 years [22]; >27 months [20]; >4.5 years [17]; 58 months [23]; 5.5 years [16]; and a minimum of 6 years [21] were all used by 1 paper each, whereas a duration of 5 years was used in 4 papers [12, 13, 24, 25]. In 4 papers, the duration of the study period was not clearly stated [11, 14, 15, 19].

The definition of late was stated as >3 months/12 weeks/90 days in 5 papers [11, 15, 18, 19, 22] and as >1 month, >2 months, >6 months and >12 months in 3 [16, 20, 21], 1 [10], 1 [13] and 1 [17] paper, respectively. The definition of late was not specified in 5 papers [12, 14, 23–25].

### Quality of evidence

Quality assessment using the CASP tool found all studies involving qualitative methods provided a clear statement of aims and study methodology, and the methods were deemed appropriate to address the aims of the research (Supplementary Material 5).

### Quantitative synthesis: meta-analysis

First, the meta-analysis tested whether the proportion of late presentation was significantly different from 0.0 (zero) for each of the strategies, and then tested the difference in the proportion of late presentation between the two strategies. Second, the meta-analysis tested the time effect, i.e. the difference between late presentation defined as less than and defined as more than 3 months of age, regardless of screening strategy.

**Fig. 2:**
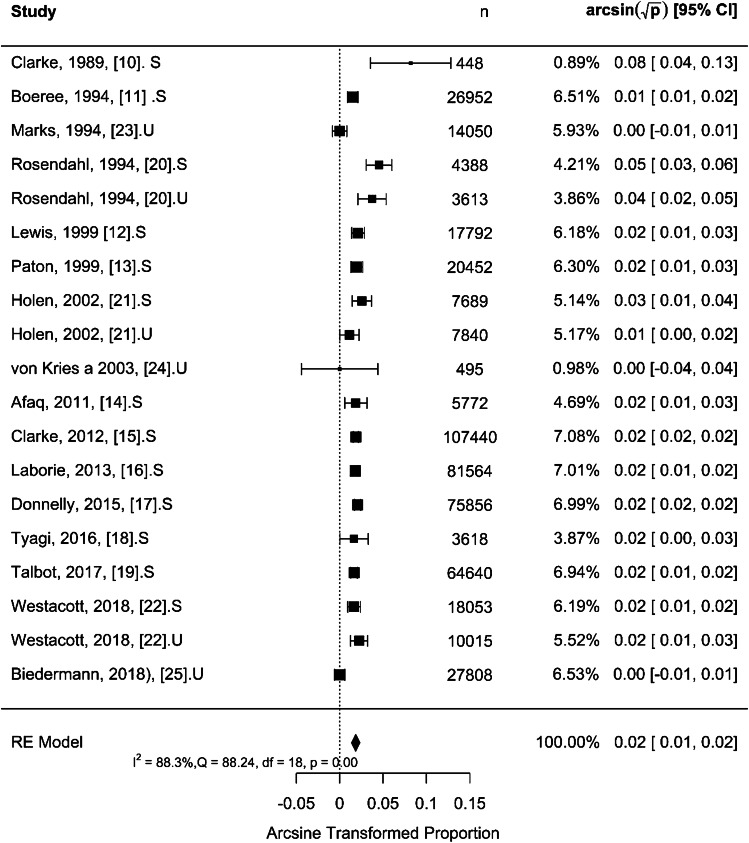
Forest plot and meta-analysis for effect size using arcsine transformed proportion of late presentation for both selective (S) and universal (U) ultrasound screening strategies. CI, confidence interval; df, degree of freedom; *I*^2^, statistics for heterogeneity; n, number; Q, test assessing heterogeneity of effect sizes; RE, random effect

The results for all data sets from 16 publications, including 19 study periods, for selective and universal US screening strategies, using arcsine-based transformations, are presented in Fig. 2. The model heterogeneity (*I*^2^) reached 88.3%, indicating high heterogeneity which is significant (*Q* = 88.24, *P* < 0.001). The overall proportion of late presentation for both selective and universal strategies combined was 0.00033 [95% CI: 0.00019–0.00053], corresponding to a rate of late presentation of 0.33 per 1,000 children [95% CI: 0.19–0.53], which was statistically significant (*P* < 0.001). This means that the proportion of late detected cases in a newborn population screened with ultrasound (universally or selectively taken together) is significantly different from zero (which would correspond to a population without any late detected cases). The Rosenthal’s method for assessing publication bias indicated that the likelihood of publication bias in our meta-analysis was minimal (*P* < 0.001).

**Fig. 3:**
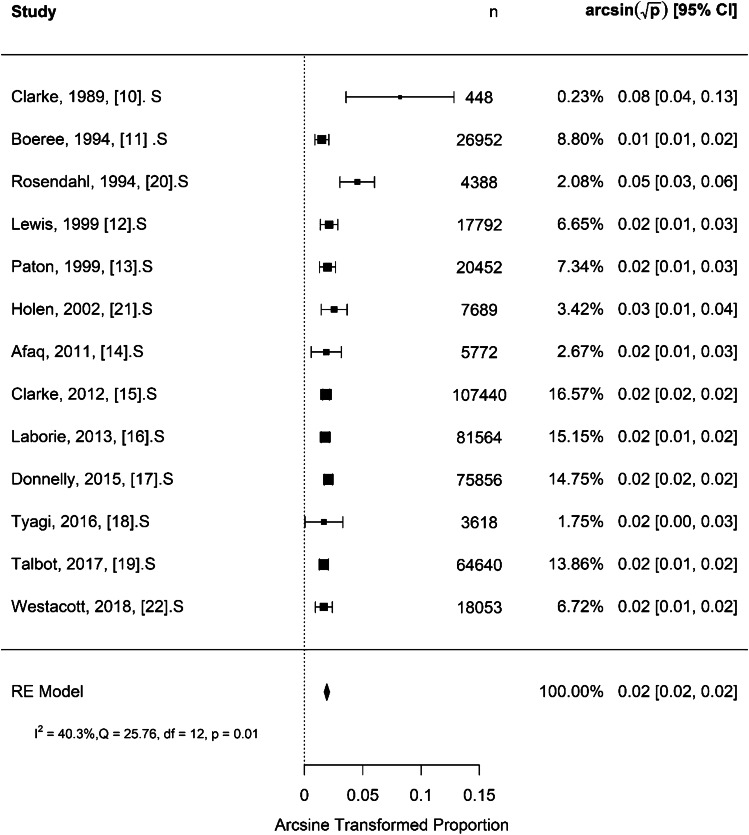
Forest plot for effect size using arcsine transformed proportion of late presentation for selective ultrasound screening strategy. CI, confidence interval; df, degree of freedom; *I*^2^, statistics for heterogeneity; n, number; Q, test assessing heterogeneity of effect sizes; RE, random effect

**Fig. 4:**
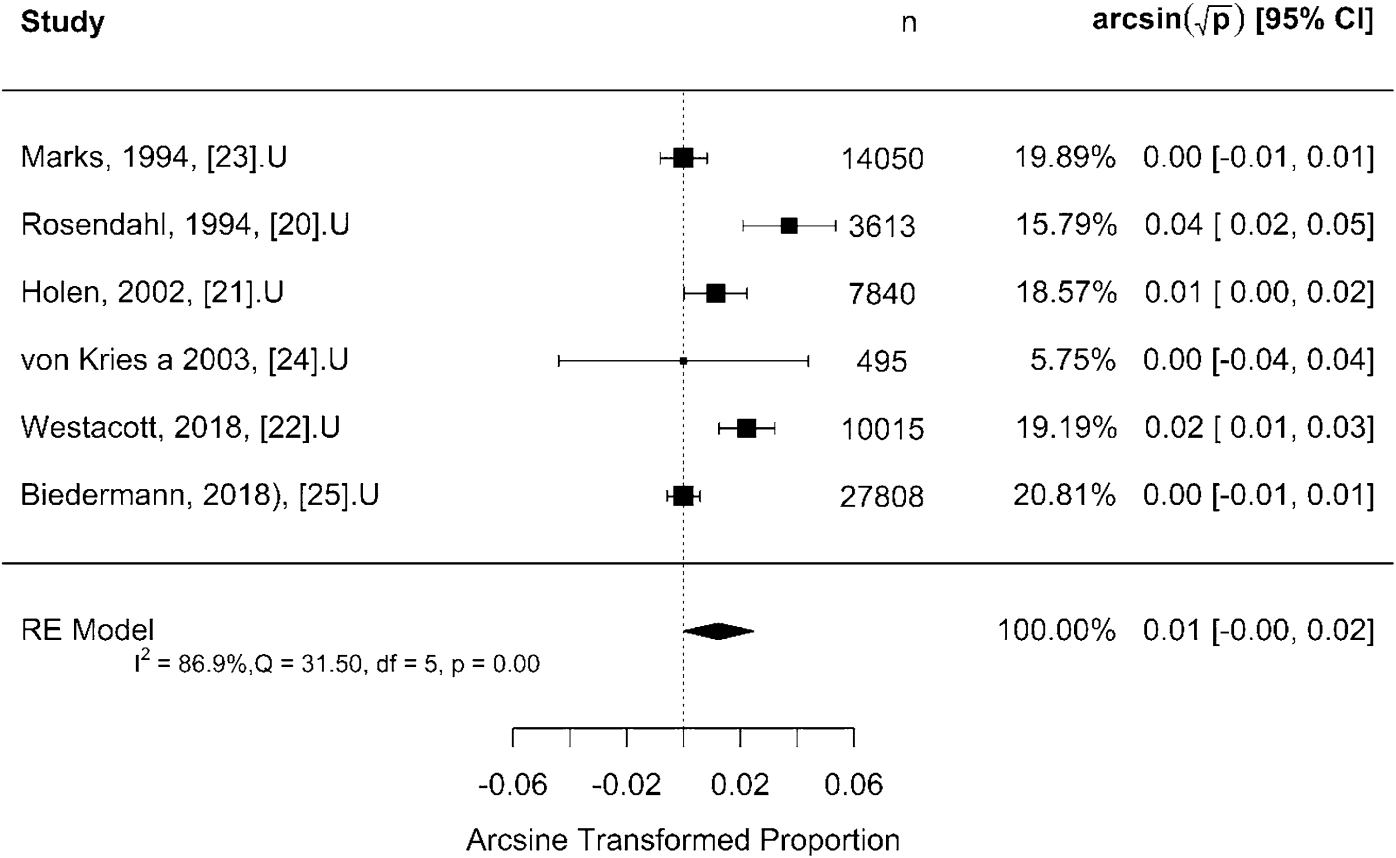
Forest plot for effect size using arcsine transformed proportion of late presentation for universal ultrasound screening strategy. CI, confidence interval; df, degree of freedom; *I*^2^, statistics for heterogeneity; n, number; Q, test assessing heterogeneity of effect sizes; RE, random effect

For selective US screening, the results shown in Fig. 3 indicate a moderate to low total heterogeneity (*I*^2^) of 40.3% which is significant (*Q* = 25.76, *P* = 0.001). The rate of late presentation per 1,000 children was 0.37 [95% CI: 0.29–0.46] (*P* < 0.001), indicating that the proportion of late detected cases in the selectively screened group is significantly different from zero. For universal US screening, Fig. 4 shows a high total heterogeneity (*I*^2^) of 86.9%. The rate of late presentation was 0.15 per 1,000 children [95% CI: 0.00–0.60] (*P* = 0.051), indicating that the proportion of late detected cases in the universally screened group is not significantly different from zero. The moderation effect was used to test the difference in proportion of late detected cases between the selective and universal US screening strategies. The difference was 0.09 per 1,000 children (*P* = 0.047).

Second, the meta-analysis tested the time effect, i.e. the difference between late presentation defined as less than 3 months of age (‘late <3 months of age’) and defined as more than 3 months of age (‘late >3 months of age’), regardless of selective or universal US screening strategy. Of the 19 study periods, 11 explicitly stated their definition of a late presentation and were thus included in this analysis [10, 11, 13, 15–22].

**Fig. 5:**
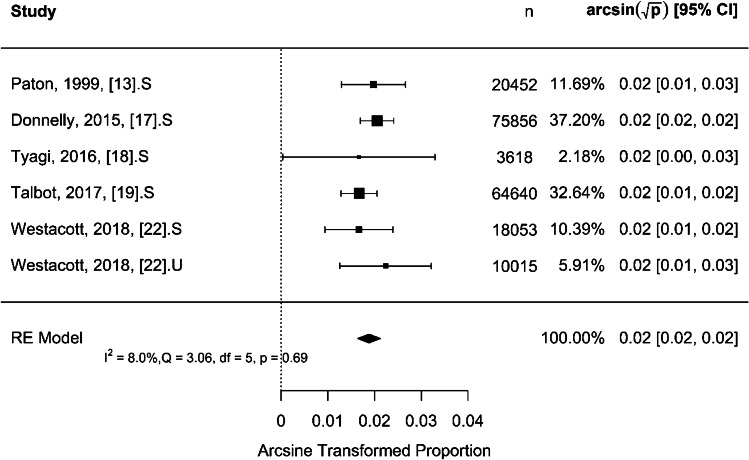
Forest plot for effect size using arcsine transformed proportion of ‘late’ presentation defined as less than 3 months of age. CI, confidence interval; df, degree of freedom; *I*^2^, statistics for heterogeneity; n, number; Q, test assessing heterogeneity of effect sizes; RE, random effect

**Fig. 6:**
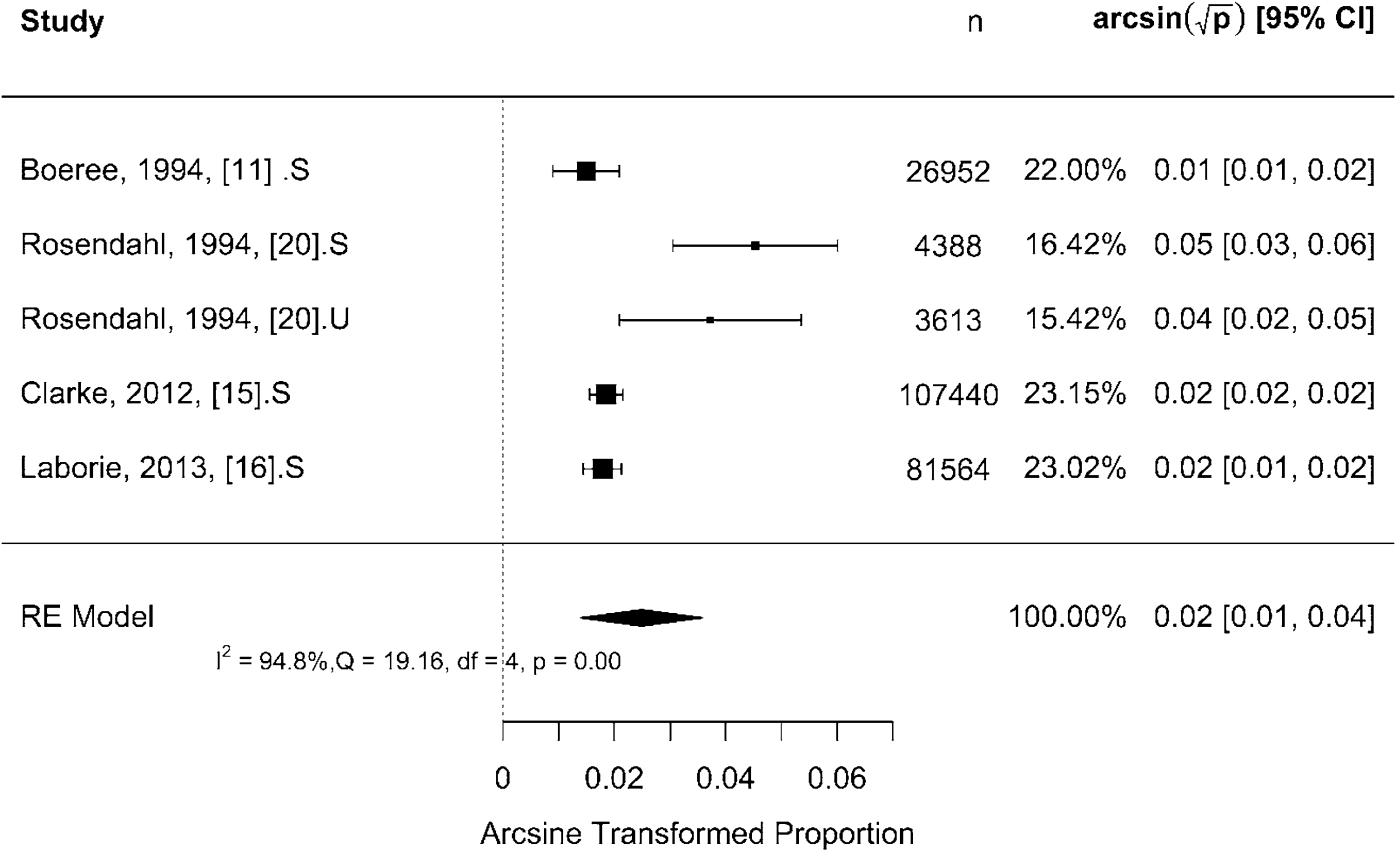
Forest plot for effect size using arcsine transformed proportion of ‘late’ presentation defined as presenting at or after 3 months of age. CI, confidence interval; df, degree of freedom; *I*^2^, statistics for heterogeneity; n, number; Q, test assessing heterogeneity of effect sizes; RE, random effect

For (‘late >3 months of age’), Fig. 5 shows that the rate of late presentation was 0.35 per 1,000 children [95% CI: 0.27–0.45] (*P* < 0.001). The total heterogeneity (*I*^2^) was 8%, which is very low and insignificant (*Q* = 3.06, *P* = 0.69). For (‘late <3 months of age’), Fig. 6 shows that the rate of late presentation was 0.62 per 1,000 children [95% CI: 0.19–1.30] (*P* < 0.001). The total heterogeneity (*I*^2^) was 95%, which is very high and significant (*Q* = 19.16, *P* < 0.001). The difference in proportion of late presentation between the different definitions of ‘late’ presentation as more than or less than 3 months of age, respectively, was tested by using the moderation effect. The difference was 0.0006 per 1,000 children (*P* = 0.272).

## Discussion

The aim of this systematic review and meta-analysis was to assess the effect of newborn selective US screening for DDH on the incidence of late presentation in infants and children, compared to a universal US strategy. The meta-analysis tested whether the proportion of late presentation was significantly different between the selective and universal US strategies. The difference was 0.09 per 1,000 children, just reaching statistical significance (*P* = 0.047), indicating a trend towards greater rate of late presentation for selective compared to universal US screening. The time effect, i.e. the difference between late presentation defined respectively, as less than and more than 3 months of age, regardless of screening strategy, was not significant (*P* = 0.272).

The quality of the evidence was generally good. We applied a rigorous methodology for the systematic review and meta-analysis. A major methodological limitation includes the large variation in definition of late presentation, and the fact that while some institutions performed newborn screening up until 6 weeks of age, other institutions classified cases detected after 4 weeks of age as ‘late detected cases’. Another limitation is the variable nature of selective US screening strategies. Reasons for this variability relate to eligibility criteria/risk factors for DDH, age at time of US screening, prevalence of DDH in each area and experience of those performing clinical hip exams and/or hip US. This variability between studies represents a limitation of the current systematic review. There was a relatively low number of studies with a universal US strategy, and these were heterogeneous, as indicated by a high *I*^2^. Several of the studies included were retrospective, which might represent a higher risk of selection bias. Limitations also include missing information or poor definition for some of the variables. Information relating to age range for clinical screening, method of clinical screening and age range for US screening were not available in 11, 7 and 10 out of the 16 papers, respectively. The follow-up period was not clear in 4 of the 16 papers and the definition of late presentation was lacking in 5 of the 16 papers. The fact that none of the included studies was performed outside Europe might affect the generalisability of the results of the meta-analysis, and thus represents a potential limitation. Lastly, no cost-effectiveness analysis has been performed.

The extensive literature relating to DDH screening in newborns reflects the lack of consensus on the topic. The studies are often difficult to compare, as the screening programmes differ substantially in the choice of strategy; in the choice of US method and in the experience of examiners (if US is performed); in definitions of DDH; in the age of the infant at time of screening; and in definitions of outcome measures for the screening programmes, which mostly include rates of late detected cases or rates of cases in need of surgical treatment. In particular, the wide variation in the age definition of a ‘late detected case’ has both clinical and research implications, and a single internationally agreed definition for ‘late’ is needed. Several systematic reviews and meta-analyses have attempted to provide recommendations for optimal screening strategy. A systematic review performed by Woolacott et al in 2005, assessing the accuracy and effectiveness of universal US screening, concluded that clear evidence was lacking either for or against general US screening of newborn infants for DDH [3]. A systematic literature review for the United States Preventive Services Task Force published in 2006 concluded that although newborns at increased risk for DDH can be identified by screening with clinical examination or ultrasound, the net benefits of screening for DDH were not clear, due to the high rates of spontaneous resolution of neonatal hip instability and dysplasia and the lack of evidence of the effectiveness of intervention on functional outcomes [4].

Similarly, a comprehensive systematic review and meta-analysis published in 2011 assessing the effect of different screening programmes for DDH on the incidence of late presentation found insufficient evidence to give clear recommendations for practice [5]. They concluded that neither of the US strategies have been demonstrated to improve clinical outcomes including late-diagnosed DDH and surgery. They stated that the studies were underpowered and thus not able to detect significant differences in rates of late detected DDH or surgery. Furthermore, they found that there was inconsistent evidence that universal US results in a significant increase in treatment compared to the use of targeted US or clinical examination alone.

Jung et al performed a systematic review and meta-analysis in 2020, assessing the effects of universal US and selective US screening on the incidence of late-diagnosed DDH [31]. To increase the power of the meta-analysis, both RCTs and cohort studies were included. They included five studies in total, without heterogeneity between studies, comprising a total of 29,070 infants screened by universal US and 30,442 infants screened by selective US. Based on their findings, the authors suggested that a statistically significant decrease in the incidence of late-diagnosed DDH is possible when universal US screening is adopted compared with selective screening. A sub-group analysis of the two RCTs showed no significant difference in late-diagnosed DDH (OR 0.52, 95% CI 0.20–1.39) for universally screened (*n* = 11,453) and selectively screened (*n* = 12,077) infants, whereas a sub-group analysis of the included cohort studies showed a significant difference in late-diagnosed DDH (OR 0.38, 95% CI 0.17–0.89) between universally screened (*n* = 17,617) and selectively screened (*n* = 18,345) infants. The meta-analysis by Jung et al included the two same RCTs as this present meta-analysis. However, the authors only included cohort studies where universal and selective US screening strategies were compared, resulting in only 3 studies. In contrast, we chose to include cohort studies reporting on only one of the strategies, and therefore have included a larger number of cohort studies and of study individuals. Similar to the findings of Jung et al, our findings indicate that selective US screening tends to increase the rate of late presentation as compared to universal US screening. However, given the small difference, and the relatively low overall incidence of late DDH (0.33 per 1,000), one might question whether offering hip US to all infants instead of those 15% at risk warrants the added costs and effort.

Cheok et al. recently published a meta-analysis examining the prevalence of late cases of DDH, with secondary outcomes being abduction bracing treatment and surgical procedures [32]. The study was performed following the implementation of universal US screening, which was compared to selective US screening programmes. They found that universal US screening showed a trend towards lower prevalence of late DDH compared to selective screening. They also found that universal screening was associated with a significant increase in the prevalence of abduction treatment without a significant reduction in the prevalence of performed surgical procedures in childhood for DDH. The authors concluded that high-quality studies on the natural history of missed DDH and on treatment methods are needed. The authors warned that their results need to be interpreted with caution as there was a lack of reliable measures to assess the incidence of late DDH, particularly for those selectively screened. Of note is that only 8 of our 16 included studies matched their 31 studies. This may in part reflect our more stringent inclusion criteria.

In summary, these meta-analyses provide important insight and underscore the need for additional cost-effectiveness analyses to update current recommendations.

## Conclusion

Selective US screening appears to slightly increase the rate of late presentation compared to universal US screening for DDH, although universal screening does not eliminate the risk of late presentation. However, uniformity in design and in reporting of DDH studies is required particularly in relation to age at clinical and US screening, to method of clinical and US screening, to duration of follow-up and to the definition of late presentation. Furthermore, a cost-effectiveness analysis is needed to update current recommendations.

## Supplementary Information

Below is the link to the electronic supplementary material.

Supplementary file1 (DOCX 14 KB)

Supplementary file2 (PDF 239 KB)

Supplementary file3 (PDF 340 KB)

Supplementary file4 (DOCX 28 KB)

Supplementary file5 (DOCX 27 KB)

## Data Availability

The datasets generated during and/or analysed during the current study are available from the corresponding author on reasonable request.
