# Supplementary material for: The effect of selective ultrasound screening on the incidence of late presentation of developmental hip dysplasia—a meta-analysis
Source: Pediatr Radiol. 2023 Apr 26;53(10):1977–88. doi: 10.1007/s00247-023-05666-x (PMC10497659; doi:10.1007/s00247-023-05666-x)
Supplement: Supplementary file 1 — Supplementary file1 (DOCX 14 KB) [file 247_2023_5666_MOESM1_ESM.docx]

**Search Strategy (9^TH^ March 2021)**

| **#** | **Database** | **Search term** | **Results** |
| --- | --- | --- | --- |
| 1 | EMBASE | "HIP DYSPLASIA"/ | 6678 |
| 3 | EMBASE | ("developmental dysplasia of the hip").ti,ab | 2533 |
| 11 | EMBASE | ("developmental hip dysplasia").ti,ab | 346 |
| 12 | EMBASE | (1 OR 3 OR 11) | 7582 |
| 2 | EMBASE | "NEWBORN SCREENING"/ | 19832 |
| 33 | EMBASE | (screening).ti,ab | 777402 |
| 35 | EMBASE | (2 OR 33) | 781966 |
| 4 | EMBASE | ("late presentation*").ti,ab | 4409 |
| 7 | EMBASE | "EARLY DIAGNOSIS"/ | 111391 |
| 8 | EMBASE | ("late sequelae").ti,ab | 1988 |
| 34 | EMBASE | "DELAYED DIAGNOSIS"/ | 13781 |
| 36 | EMBASE | (4 OR 7 OR 8 OR 34) | 130023 |
| 37 | EMBASE | (12 AND 35 AND 36) | 128 |
| 19 | Medline | "HIP DISLOCATION, CONGENITAL"/ | 7891 |
| 15 | Medline | ("developmental dysplasia of the hip").ti,ab | 2582 |
| 16 | Medline | ("developmental hip dysplasia").ti,ab | 267 |
| 17 | Medline | ("late presentation*").ti,ab | 2875 |
| 21 | Medline | "EARLY DIAGNOSIS"/ | 27554 |
| 18 | Medline | ("late sequelae").ti,ab | 1757 |
| 30 | Medline | "DELAYED DIAGNOSIS"/ OR "UNDIAGNOSED DISEASES"/ | 6878 |
| 20 | Medline | "NEONATAL SCREENING"/ | 10495 |
| 25 | Medline | (screening).ti,ab | 548364 |
| 27 | Medline | "MASS SCREENING"/ | 106362 |
| 22 | Medline | (19 OR 15 OR 16) | 8958 |
| 28 | Medline | (20 OR 25 OR 27) | 583901 |
| 31 | Medline | (17 OR 21 OR 18 OR 30) | 38636 |
| 32 | Medline | (22 AND 28 AND 31) | 85 |

**Contents** 128 of 128 results on EMBASE - (("HIP DYSPLASIA"/ OR (developmental dysplasia of the hip).ti,ab OR (developmental hip dysplasia).ti,ab) AND ("NEWBORN SCREENING"/ OR (screening).ti,ab)) AND ((late presentation*).ti,ab OR "EARLY DIAGNOSIS"/ OR (late sequelae).ti,ab OR "DELAYED DIAGNOSIS"/)

**Contents** 85 of 85 results on Medline - (("HIP DISLOCATION, CONGENITAL"/ OR (developmental dysplasia of the hip).ti,ab OR ("developmental hip dysplasia").ti,ab) AND ("NEONATAL SCREENING"/ OR (screening).ti,ab OR "MASS SCREENING"/)) AND (("late presentation*").ti,ab OR "EARLY DIAGNOSIS"/ OR ("late sequelae").ti,ab OR "DELAYED DIAGNOSIS"/ OR "UNDIAGNOSED DISEASES"/)
