# Supplementary material for: The effect of selective ultrasound screening on the incidence of late presentation of developmental hip dysplasia—a meta-analysis
Source: Pediatr Radiol. 2023 Apr 26;53(10):1977–88. doi: 10.1007/s00247-023-05666-x (PMC10497659; doi:10.1007/s00247-023-05666-x)
Supplement: Supplementary file 2 — Supplementary file2 (PDF 239 KB) [file 247_2023_5666_MOESM2_ESM.pdf]

CASP
Critical Appraisal Skills Programme

www.casp-uk.net
info@casp-uk.net
Summertown Pavilion, Middle Way Oxford OX2 7LG

CASP Checklist: 12 questions to help you make sense of a Cohort Study

How to use this appraisal tool: Three broad issues need to be considered when appraising a cohort study:

- Are the results of the study valid? (Section A)
- What are the results? (Section B)
- Will the results help locally? (Section C)

The 12 questions on the following pages are designed to help you think about these issues systematically. The first two questions are screening questions and can be answered quickly. If the answer to both is "yes", it is worth proceeding with the remaining questions. There is some degree of overlap between the questions, you are asked to record a "yes", "no" or "can't tell" to most of the questions. A number of italicised prompts are given after each question. These are designed to remind you why the question is important. Record your reasons for your answers in the spaces provided.

About: These checklists were designed to be used as educational pedagogic tools, as part of a workshop setting, therefore we do not suggest a scoring system. The core CASP checklists (randomised controlled trial & systematic review) were based on JAMA 'Users' guides to the medical literature 1994 (adapted from Guyatt GH, Sackett DL, and Cook DJ), and piloted with health care practitioners.

For each new checklist, a group of experts were assembled to develop and pilot the checklist and the workshop format with which it would be used. Over the years overall adjustments have been made to the format, but a recent survey of checklist users reiterated that the basic format continues to be useful and appropriate.

Referencing: we recommend using the Harvard style citation, i.e.: *Critical Appraisal Skills Programme (2018). CASP (insert name of checklist i.e. Cohort Study) Checklist. [online] Available at: URL. Accessed: Date Accessed.*

©CASP this work is licensed under the Creative Commons Attribution – Non-Commercial-Share A like. To view a copy of this license, visit http://creativecommons.org/licenses/by-nc-sa/3.0/ www.casp-uk.net

CASP
Critical Appraisal
Skills Programme

Paper for appraisal and reference:...

Section A: Are the results of the study valid?

1. Did the study address a clearly focused issue?

Yes ☐
Can't Tell ☐
No ☐

HINT: A question can be 'focused' in terms of
- the population studied
- the risk factors studied
- is it clear whether the study tried to detect a beneficial or harmful effect
- the outcomes considered

Comments:

2. Was the cohort recruited in an acceptable way?

Yes ☐
Can't Tell ☐
No ☐

HINT: Look for selection bias which might compromise the generalisability of the findings:
- was the cohort representative of a defined population
- was there something special about the cohort
- was everybody included who should have been

Comments:

Is it worth continuing?

CASP
Critical Appraisal
Skills Programme

3. Was the exposure accurately measured to minimise bias?

Yes ☐
Can't Tell ☐
No ☐

HINT: Look for measurement or classification bias:
- did they use subjective or objective measurements
- do the measurements truly reflect what you want them to (have they been validated)
- were all the subjects classified into exposure groups using the same procedure

Comments:

4. Was the outcome accurately measured to minimise bias?

Yes ☐
Can't Tell ☐
No ☐

HINT: Look for measurement or classification bias:
- did they use subjective or objective measurements
- do the measurements truly reflect what you want them to (have they been validated)
- has a reliable system been established for detecting all the cases (for measuring disease occurrence)
- were the measurement methods similar in the different groups
- were the subjects and/or the outcome assessor blinded to exposure (does this matter)

Comments:

5. (a) Have the authors identified all important confounding factors?

Yes ☐
Can't Tell ☐
No ☐

HINT:
- list the ones you think might be important, and ones the author missed

Comments:

5. (b) Have they taken account of the confounding factors in the design and/or analysis?

Yes ☐
Can't Tell ☐
No ☐

HINT:
- look for restriction in design, and techniques e.g. modelling, stratified-, regression-, or sensitivity analysis to correct, control or adjust for confounding factors

Comments:

6. (a) Was the follow up of subjects complete enough?

Yes ☐
Can't Tell ☐
No ☐

HINT: Consider
- the good or bad effects should have had long enough to reveal themselves
- the persons that are lost to follow-up may have different outcomes than those available for assessment
- in an open or dynamic cohort, was there anything special about the outcome of the people leaving, or the exposure of the people entering the cohort

6. (b) Was the follow up of subjects long enough?

Yes ☐
Can't Tell ☐
No ☐

Comments:

Section B: What are the results?

7. What are the results of this study?

HINT: Consider
- what are the bottom line results
- have they reported the rate or the proportion between the exposed/unexposed, the ratio/rate difference
- how strong is the association between exposure and outcome (RR)
- what is the absolute risk reduction (ARR)

Comments:

8. How precise are the results?

HINT:
- look for the range of the confidence intervals, if given

Comments:

9. Do you believe the results?

Yes ☐
Can't Tell ☐
No ☐

HINT: Consider
- big effect is hard to ignore
- can it be due to bias, chance or confounding
- are the design and methods of this study sufficiently flawed to make the results unreliable
- Bradford Hills criteria (e.g. time sequence, dose-response gradient, biological plausibility, consistency)

Comments:

Section C: Will the results help locally?

10. Can the results be applied to the local population?

Yes ☐
Can't Tell ☐
No ☐

HINT: Consider whether
- a cohort study was the appropriate method to answer this question
- the subjects covered in this study could be sufficiently different from your population to cause concern
- your local setting is likely to differ much from that of the study
- you can quantify the local benefits and harms

Comments:

11. Do the results of this study fit with other available evidence?

Yes ☐
Can't Tell ☐
No ☐

Comments:

Critical Appraisal Skills Programme

12. What are the implications of this study for practice?

Yes ☐

Can't Tell ☐

No ☐

HINT: Consider
- one observational study rarely provides sufficiently robust evidence to recommend changes to clinical practice or within health policy decision making
- for certain questions, observational studies provide the only evidence
- recommendations from observational studies are always stronger when supported by other evidence

Comments:
