# Supplementary material for: The effect of selective ultrasound screening on the incidence of late presentation of developmental hip dysplasia—a meta-analysis
Source: Pediatr Radiol. 2023 Apr 26;53(10):1977–88. doi: 10.1007/s00247-023-05666-x (PMC10497659; doi:10.1007/s00247-023-05666-x)
Supplement: Supplementary file 4 — Supplementary file4 (DOCX 28 KB) [file 247_2023_5666_MOESM4_ESM.docx]

Supplementary Material 4: Data Extraction Sheet

Each paper was assigned to one of the researchers, and data was extracted and entered in an Excel spreadsheet.

| GENERAL: |
| --- |
| Co-Researcher |
| Paper |
| Country of paper |
| Type of study |
| Total population (in study period) |
| Total screened by ultrasound |
| Included in the study (number) |
| Selective screening rate (%) |
| Number female patients screened by ultrasound |
| % female patients screened by ultrasound |
| Youngest age at clinical screening |
| Oldest age at clinical screening |
| Youngest age at ultrasound screening |
| Oldest age at ultrasound screening |
| Role/experience clinician |
| Clinical screening method(s) |
| Role/experience person scanning |
| Ultrasound method |
| Abnormal or equivocal clinical findings (number) |
| Selective or non-selectve screening |
| Positive family history (number) |
| Breech (number) |
| Foot deformities |
| Torticollis |
| Other 1 (please specifiy) |
| Other 2 |
| Other 3 |
| Definition of Late |
| Duration of follow-up/study |
| Comments |
| RESULTS |
| Number of patients with abnormal ultrasounds |
| Number of ultrasounds performed |
| % abnormal ultrasound scans |
| Number females with abnormal ultrasounds |
| % females with abnormal ultrasounds |
| Total number clinically abnormal hips |
| Number clinically abnormal hips with abnormal ultrasound |
| % clinically abnormal hips with abnormal ultrasound |
| Number of abnormal ultrasounds with risk factor |
| % abnormal ultrasounds with risk factor |
| Number treated in a pavlik harness |
| Harness treatment rate (per 1000) |
| Number treated with a "cotton napkin" until Graf type I had been reached |
| Cotton napkin treatment % |
| Number of surgically treated cases |
| Surgical treatment rate (per 1000) |
| Closed reduction rate (per 1000) |
| Open reduction rate (per 1000) |
| Number late cases |
| Rate of late cases (per 1000) |
| Youngest age late diagnosis (months) |
| Oldest age late diagnosis (months) |
| Number AVN |
| Rate of AVN |
| Other complication 1 |
| Number other 1 |
| Rate other 1 |
| Comments |
|  |
