# Supplementary material for: The effect of selective ultrasound screening on the incidence of late presentation of developmental hip dysplasia—a meta-analysis
Source: Pediatr Radiol. 2023 Apr 26;53(10):1977–88. doi: 10.1007/s00247-023-05666-x (PMC10497659; doi:10.1007/s00247-023-05666-x)
Supplement: Supplementary file 5 — Supplementary file5 (DOCX 27 KB) [file 247_2023_5666_MOESM5_ESM.docx]

Table 2: Quality assessment of the included studies. Quality assessment was performed using the critical appraisal skills programme check lists for cohort studies and randomised controlled trials

| First author (year) | | 1. Did the study address a clearly  focused issue? | | 2. Was the cohort recruited in an acceptable way? | | 3. Was the exposure accurately measured to minimise bias? | | 4. Was the outcome accurately measured to minimise bias? | | 5. (a) Have the authors identified all important confounding factors? | | 5. (b) Have they taken account of the confounding factors in the design and/or analysis? | | | 6. (a) Was the follow up of subjects complete enough? | | | 6. (b) Was the follow up of subjects long enough? | | | 9. Do you believe the results? | | 10. Can the results be applied to the local population? | | | 11. Do the results of this study fit with other available evidence? | | | 12. What are the implications of this study for practice? | | |  |  |  |  |  |  |  |  |  |  |  |  |  |
| --- | --- | --- | --- | --- | --- | --- | --- | --- | --- | --- | --- | --- | --- | --- | --- | --- | --- | --- | --- | --- | --- | --- | --- | --- | --- | --- | --- | --- | --- | --- | --- | --- | --- | --- | --- | --- | --- | --- | --- | --- | --- | --- | --- | --- |
|  | |  | |  | |  | |  | |  | |  | | |  | | |  | | |  | |  | | |  | | |  | | |  | | |  | | |  | | |  | |  |  |
| **Cohort studies:** | | | |  | |  | |  | |  | |  | | |  | | |  | | |  | |  | | |  | | |  | | |  | | |  | | |  | | |  | |  |  |
| Clarke, 1989 [10] | | Y | | Y | | Y | | Y | | C | | C | | | Y | | | Y | | | Y | | Y | | | C | | | C | | |  | | |  | | |  | | |  | |  |  |
| Boeree, 1994 [11] | | Y | | Y | | C | | Y | | Y | | Y | | | Y | | | Y | | | Y | | Y | | | Y | | | Y | | |  | | |  | | |  | | |  | |  |  |
| Marks, 1994 [23] | | Y | | Y | | C | | Y | | C | | C | | | Y | | | Y | | | Y | | Y | | | Y | | | Y | | |  | | |  | | |  | | |  | |  |  |
| Lewis, 1999 [12] | | Y | | Y | | C | | N | | C | | C | | | Y | | | Y | | | C | | Y | | | Y | | | Y | | |  | | |  | | |  | | |  | |  |  |
| Paton, 1999 [13] | | Y | | Y | | C | | Y | | N | | N | | | C | | | Y | | | Y | | Y | | | Y | | | Y | | |  | | |  | | |  | | |  | |  |  |
| von Kries, 2003 [24] | | Y | | Y | | Y | | Y | | Y | | Y | | | Y | | | Y | | | Y | | Y | | | Y | | | Y | | |  | | |  | | |  | | |  | |  |  |
| Afaq, 2011 [14] | | Y | | Y | | Y | | Y | | C | | C | | | Y | | | Y | | | Y | | Y | | | Y | | | C | | |  | | |  | | |  | | |  | |  |  |
| Clarke, 2012 [15] | | Y | | Y | | Y | | Y | | Y | | Y | | | Y | | | Y | | | Y | | Y | | | Y | | | Y | | |  | | |  | | |  | | |  | |  |  |
| Laborie, 2013 [16] | | Y | | Y | | Y | | Y | | Y | | Y | | | Y | | | Y | | | Y | | Y | | | Y | | | Y | | |  | | |  | | |  | | |  | |  |  |
| Donnelly, 2015 [17] | | Y | | Y | | Y | | Y | | Y | | Y | | | Y | | | Y | | | Y | | Y | | | Y | | | Y | | |  | | |  | | |  | | |  | |  |  |
| Tyagi, 2016 [18] | | C | | Y | | C | | Y | | C | | C | | | Y | | | Y | | | C | | Y | | | Y | | | Y | | |  | | |  | | |  | | |  | |  |  |
| Talbot, 2017 [19] | | Y | | Y | | Y | | C | | Y | | N | | | Y | | | Y | | | Y | | Y | | | Y | | | Y | | |  | | |  | | |  | | |  | |  |  |
| Westacott, 2018 [22] | | Y | | C | | Y | | Y | | Y | | Y | | | Y | | | Y | | | Y | | Y | | | Y | | | Y | | |  | | |  | | |  | | |  | |  |  |
| Biedermann, 2018 [25] | | Y | | Y | | C | | C | | Y | | Y | | | Y | | | Y | | | Y | | Y | | | C | | | Y | | |  | | |  | | |  | | |  | |  |  |
|  | |  | |  | |  | |  | |  | |  | | |  | | |  | | |  | |  | | |  | | |  | | |  | | |  | | |  | | |  | |  |  |
|  |  | |  | |  | |  | |  | |  | | |  | | |  | | |  | | | |  | | |  | | |  | | | |  | | |  | | |  | | | |  |
| Author (year) | 1. Did the study address a clearly focused research question? | | 2. Was the assignment of participants to interventions randomised? | | 3. Were all participants who entered the study accounted for at its conclusion? | | 4. Were the participants ‘blind’ to intervention they were given? | | Were the investigators ‘blind’ to the intervention they were giving to participants? | | Were the people assessing/ analysing outcome/s ‘blinded’? | | 5. Were the study groups similar at the start of the randomised controlled trial? | | | 6. Apart from the experimental intervention, did each study group receive the same level of care (that is, were they treated equally)? | | | 9. Do the benefits of the experimental intervention outweigh the harms and costs? | | | 10. Can the results be applied to your local population/ in your context? | | | 11. Would the experimental intervention provide greater value to the people in your care than any of the existing interventions? | | | | | |  |  |  |  |  |  |  |  |  |  |  |  |  |  |
| **Randomised controlled trials:** | | |  | |  | |  | |  | |  | |  | | |  | | |  | | |  | | |  | | | | | |  |  |  |  |  |  |  |  |  |  |  |  |  |  |
| Rosendahl 1994 [20] | Y | | Y | | Y | | N | | N | | Y | | Y | | | Y | | | C | | | Y | | | Y | | |  | | |  | |  | | |  | | |  | | |  |  |  |
| Holen, 2002 [21] | Y | | Y | | N | | N | | N | | N | | N | | | Y | | | N | | | Y | | | N | | |  | | |  | |  | | |  | | |  | | |  |  |  |
|  |  | |  | |  | |  | |  | |  | | |  | | |  | | |  | | | |  | | |  | | |  | | | |  | | |  | | |  | | | |  |
|  |  | |  | |  | |  | |  | |  | | |  | | |  | | |  | | | |  | | |  | | |  | | | |  | | |  | | |  | | | |  |

*C* cannot tell, *N* no, *Y* yes
